# Hexagonal Nano and Micro Boron Nitride: Properties and Lubrication Applications

**DOI:** 10.3390/ma15030955

**Published:** 2022-01-26

**Authors:** Szymon Senyk, Arkadiusz Chodkiewicz, Krzysztof Gocman, Barbara Szczęśniak, Tadeusz Kałdoński

**Affiliations:** 1 Doctoral School, Military University of Technology, 00-908 Warsaw, Poland; 2 Faculty of Mechanical Engineering, Military University of Technology, 00-908 Warsaw, Poland; krzysztof.gocman@wat.edu.pl (K.G.); tadeusz.kaldonski@wat.edu.pl (T.K.); 3 Institute of Chemistry, Military University of Technology, 00-908 Warsaw, Poland; barbara.szczesniak@wat.edu.pl

**Keywords:** hexagonal boron nitride, tribology, nanoparticles, lubricant additives, solid lubricants

## Abstract

The article presents a discussion on the use of hexagonal boron nitride as an additive to lubricants. Based on the analysis of the research, factors determining its application were identified. These include particle size distribution, their morphology, specific surface area, and porosity. Next, the research identifying these properties for the four types of h-BN was described. Based on the results, the possible mechanisms of the influence of individual h-BN types were described. It was also found that the use of h-BN nanoparticles as lubricants seems to be promising.

## 1. Introduction

**Figure 1:**
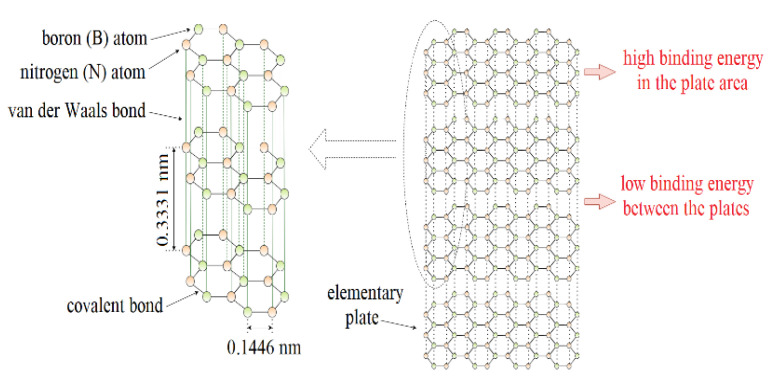
Lamellar structure of hexagonal boron nitride.

Hexagonal boron nitride (h-BN or α-BN) is one of the polymorphic forms of boron nitride. In recent years, thanks to the optimization of the h-BN production process, environmental conditions, and excellent properties, there is an increased interest in this material. It has interesting properties predisposing it to be used in lubrication applications. These include excellent chemical resistance, thermal stability, and good thermal conductivity with no electrical conductivity. From a tribological perspective, the lamellar structure of hexagonal boron nitride is particularly interesting (Figure 1). In each layer, the boron and nitrogen atoms are arranged alternately and the length of the B–N bond is ~1.45 Å (0.1446 nm). Nitrogen and boron atoms, connected by the sp^2^ orbital, form a strong s-type covalent bond. There are large interplanar distances between the adjacent layers of hexagonal boron nitride, resulting in only weak interactions between the atoms of these layers in the form of van der Waals forces with an energy value of about 16.7 kJ/mol. This translates into easy interlayer sliding and low internal friction resistance. The atomic layers align parallel to the direction of sliding motion and shear with relative ease, providing a low friction resistance [1,2,3,4,5].

Similar to other solid lubricants, such as molybdenum disulfide, tungsten disulfide, and graphite, hexagonal boron nitride is considered an additive to lubricants. Lubricants that do not contain appropriate additives do not allow to obtain the required lubrication efficiency in difficult operating conditions, such as low and high temperatures, under high pressure, at very low and high sliding speeds, in a vacuum, or in the presence of radiation. This is how the idea of introducing solid lubricants into lubricants was born. Furthermore, the fact that the additives used to date, such as heavy metals, sulfur, and phosphorus compounds, are harmful to the environment is a key factor. They can be replaced by additives in the form of solid lubricants, while increasing the tribological performance of the lubricants [6].

The complexity of the mechanisms of the tribological interaction of additives with a layered structure, resulting in an irregular variation of the coefficient of friction with the increase in the applied load, was pointed out by the authors of publications [7,8], who analyzed molybdenum disulfide. Due to the affinity in terms of structure and similar physicochemical properties, it would not be wrong to adopt such a conclusion also about h-BN. The authors of publication [7] suggest that a comprehensive research investigation is required to indicate the correctness of operations of such additives. It would include both an analysis of the properties of the additive and a detailed analysis of the tribological tests. This article focuses on the first of the indicated areas by analyzing the properties of hexagonal boron nitride, which is used as an additive to greases and lubricating oils.

## 2. Hexagonal Boron Nitride as an Additive to Lubricants

The possibility of using hexagonal boron nitride as an additive to lubricants was already tested in the 1990s. In the series of publications [4,9,10,11,12,13,14] resulting from research carried out at the Military University of Technology (Warsaw, Poland), h-BN with an average grain size of ~0.5 µm was considered an adequate additive to greases. Various tribological tests conducted on a four-ball apparatus, a KEWAT-6 friction machine, a vibrating test bench, and an analysis of the resistance to the motion of rolling bearings have shown that this additive, in certain concentrations in the range of 2–10% (by wt.), depending on the base grease and friction conditions, improves the tribological properties of the composition. Article [15] describes the research of the friction torque between the spherical surfaces of the steering rod pin and its seat. The addition of 4% h-BN (the authors did not specify the h-BN granulation in the article) to lithium grease reduced the frictional resistance by 27–50% compared to the reference sample. Lithium grease containing 1%, 3%, or 9% h-BN with a grain size of ~1 µm was tested on a four-ball apparatus and described in another publication [16]. Based on the adopted test criteria, the concentration of 1% (by wt.) was considered the best. Increasing the concentration of boron nitride above 1% was considered to be pointless as it did not improve the lubrication efficiency. On the other hand, greases which were a composition of a mineral oil thickened with lithium 12-hydroxy stearate, to which h-BN was added in concentrations of 5%, 10%, and 20%, were analyzed by other authors [17]. They used h-BN with dimensions of 0.5 μm, 5 μm, and 30 μm. The research was carried out with a reciprocating sliding motion using a ball-on-flat contact configuration. It was found that h-BN provides good lubrication conditions and better surface quality. Unlike graphite, it did not leave any dark spots on the formed surface, so no additional grinding and polishing was necessary. The best tribological performance was determined for h-BN powder with granulation of 30 μm added to the grease at a concentration of 10 to 20%. However, other authors used the addition of nano h-BN with an average grain size of 80 nm, in mass concentrations of 0.5%, 0.67%, 1.33%, 1.5%, and 2% for lithium grease [18]. The tests were carried out on a tribotester with a ball-on-disc contact configuration. The application of 2% nano h-BN resulted in a 46% lower value of the coefficient of friction than the base grease. Article [19] describes the tests of lithium grease containing h-BN with an average diameter of 449.35 nm in concentrations of 0.15%, 0.30%, 0.45%, 0.60%, 0.75%, and 0.90%. The tests carried out on a four-ball apparatus showed that the lowest value of the coefficient of friction allowed to obtain a grease containing 0.60% h-BN. Its use also resulted in the best protection of friction surfaces. However, according to the publication [20], the h-BN addition had a negative effect on the tribological properties of the grease. Low-temperature greases based on a mixture of synthetic polyalphaolefin and subacute dioctyl and thickened with modified silica, to which h-BN was added (its granulation was not determined) at concentrations of 2%, 4%, and 8%, were tested on a four-ball apparatus. The deterioration of anti-seizing and anti-wear properties was found when h-BN was added to the grease.

The possibility of using hexagonal boron nitride was also tested in the context of lubricating oils. Article [21] describes the use of this solid lubricant in a mixture with paraffinic mineral oil. Tests conducted on a ring-on-roller apparatus (bearing steel–bearing steel contact) indicated that 1% (by wt.) of h-BN significantly reduced wear. As the concentration increased, a reduction in wear was still observed. However, the coefficient of friction increased slightly. The mixtures of hexagonal boron nitride with oils were prepared at the Military University of Technology (Warsaw, Poland) and tested under the conditions mentioned in the previous paragraph [4,12,22,23]. The additions of 0.5%, 2%, and 4% h-BN were used. In the tests on the four-ball apparatus, the best results were obtained for the oil containing 2% h-BN. In the case of porous bearings impregnated with oil with hexagonal boron nitride, a significant improvement in the operational properties of the oil and an increase in the bearing load capacity were observed. Other authors [24,25,26] in a series of articles analyzed the effect of particle size on friction and wear during sliding contact on a pin-on-disc machine. During the experiment, h-BN particles (addition of 5% by wt.) of micrometer, submicrometer, and nanometer size in mixtures with rapeseed oil or avocado oil were examined. The best tribological properties (reduction in the friction coefficient and reduction in wear) were found in mixtures containing nanoadditives. It was concluded that the nanoparticles penetrate the friction area better, precisely because of their small size and spherical shape. The research also showed the influence of the surface roughness of the materials on the tribological quality. For smoother surfaces, the quality increased when smaller particles were used. For rough surfaces, a positive effect was given by larger particles. Motor oil (SAE 10W) with the addition of hexagonal boron nitride with an average grain diameter of 114 nm was tested on a ball-on-disc tribotester and presented in a subsequent publication [27]. The influence of nanoparticles on the change in the coefficient of friction and wear was observed. In the case of their application, a 14.4% reduction in the coefficient of friction and a 65% reduction in wear compared to the reference engine oil were achieved. The use of hexagonal boron nitride with a grain diameter of 50 nm in a blend with SAE 20W50 oil resulted in a significant improvement in anti-seize and anti-wear properties [28]. Mixtures with 1%, 2%, and 3% h-BN were tested. The mixture containing 3% h-BN achieved the lowest value of the coefficient of friction. In article [29], mixtures of hexagonal boron nitride with 15W-40 oil were prepared. Oleic acid (addition of 25% by weight of h-BN) was used to stabilize the suspension. Mixtures containing 0.1%, 0.5%, and 1% h-BN with an average grain diameter of ~120 nm were prepared. Compared to the base oil, the coefficient of friction decreased by 76.9%, 53.8%, and 27.7% for the tests with the addition of 0.1%, 0.5%, and 1% h-BN, respectively.

A significant problem, not observed in the case of greases, is the instability of the suspension consisting of oil and hexagonal boron nitride. The result is that the particles of this powdered material, which has a higher density than lubricating oils, sediment as a result of gravity [12]. To prevent this, researchers use chemical additives to maintain a stable suspension of the powder in the solution [29,30]. Another way may be to modify the method of preparing the mixture by applying additional procedures, such as sonication [28,31] or by shaking the samples [24,25].

The authors of publications [6,32,33] emphasize that the efficiency of lubrication is strongly dependent on the type of particles, their size, and concentration in a specific lubricant. Therefore, the few cases of deterioration of the tribological properties of the lubricant after adding solid lubricants to them result from incorrectly selecting the base lubricant, inadequately selecting the concentration or the granulation of the additive. Attention should also be paid to particle morphology. According to the authors of the publications [7,8,34], particles with sharp edges can behave in the manner of abrasives, especially at lower loads. Particles with rounded, curved edges result in lower coefficients of friction and less wear.

There is a growing interest in the addition of nanoadditives to lubricants, resulting from the dynamic development of nanotechnology. Such an application seems promising for several reasons. Scientific reports indicate that nanoparticles can penetrate the friction area more efficiently, which may reduce the wear of cooperating elements [7,18,35]. They can also fill in defects in the material on the surfaces, thus preventing their further wear [35]. It was also observed that oils containing nanoparticles reduce the mean arithmetic deviation of the lubricated surface roughness profile about the mean line [29]. Moreover, it was found that, under mixed or boundary lubrication conditions, nanoparticles can form a protective layer enabling micro polishing and surface repair [27,36,37,38].

A characteristic feature of nanomaterials is the large grain boundary area. For powder materials, such as h-BN, the separation limit is the outer surface of the particles. It translates into strong chemical reactivity and propensity for the formation of agglomerates [39]. This is of particular importance in the case of lubricating oils with the addition of nano h-BN, as it makes it difficult to obtain a stable suspension and, to obtain it, it is necessary to use additional treatments, mentioned above. The author of article [40] states that the specific surface area is considered an important property of powdered materials, as it significantly affects their physical and chemical properties. Moreover, a relationship between this parameter and the average particle size was noticed—the surface area per unit mass decreases with the increase in the average particle size. However, this is not the only factor determining the specific surface area. It is highly dependent on the porous structure of the particles. The authors of publication [19] report that the highly developed pore structure and large specific surface area can help to increase the degree of binding of the lubricant and h-BN particles.

In summary, the determinants of the effectiveness of hexagonal boron nitride include the particle size distribution and an appropriately selected concentration for a specific lubricant. The recommended parameter for describing the set of particles is precisely the particle size distribution, and not only the average particle size. In the case of materials with a large size dispersion of individual matter elements, the averaged result does not reflect the actual granulation of the sample. Identification of the particle shape, size, as well as specific surface area and porous structure is the starting point for considering its interaction with the lubricant and rubbing surfaces. Without this information, it seems unjustified to formulate theses on the mechanisms of interaction of hexagonal boron nitride particles in various tribological systems.

## 3. Experimental Methods

The research part of this article contains an analysis of the properties of four types of hexagonal boron nitride intended for application as additives to lubricating oils and greases. For this purpose, research methods were used to enable the features of h-BN, determining its effectiveness as such an additive.

### 3.1. Crystal Structure Determination—X-ray Diffraction (XRD)

The phase analysis was carried out based on the X-ray diffraction patterns obtained with an X-ray powder diffractometer Rigaku Ultima IV equipped with a cobalt anode lamp (λ = 1.78 Å) at the operating parameters of 40 mA, 40 kV and 1°/min scanning speed. Cross-beam optics (CBO), parallel-beam geometry, and a fast linear counter (Detex Ultra), which resulted in patterns with very low Kβ levels, were also used for characterization. The sample was mounted in a standard, glass, non-rotating sample holder. The analysis was performed using PDXL (Rigaku) software and the PDF4+ 2021 database.

### 3.2. Quantitative and Qualitative Assessment of the Surface Composition—X-ray Photoelectron Spectroscopy (XPS)

The analysis was performed with a PHI VersaProbeII apparatus (ULVAC-PHI, Chigasaki, Japan) using focused monochromatic X-rays from the Al Kα line (1486.6 eV). The beam was focused on a spot of 100 µm and scanned a 400 µm × 400 µm area on the sample surface. For each measurement spot, one wide-range spectrum (0–1300 eV) with low-resolution power (0.5 eV) and a high-resolution spectrum (0.1 eV) were measured in the areas of line occurrence: C 1s, O 1s, B 1s, and N 1s. During the measurements, surface charge neutralization was applied by irradiating the surface with a beam of low-energy electrons (1 eV) and ions (7 eV Ar^+^) to ensure a constant surface potential despite the emitted photoelectrons. The vacuum value during the measurement oscillated around 2 × 10^−9^ mbar. The fitting of the spectral lines was carried out using the PHI Multipak program (v.9.9.0.8) by subtracting the background using the Shirley method [41]. The measurement geometry used results in an analysis depth of 5 nm.

### 3.3. Imaging and Determination of Particle Size Distribution—Scanning Electron Microscope (SEM)

The test specimens were prepared by carefully sprinkling a small amount of the powder on a tape with carbon glue. To obtain better quality microscopic images, the specimens were coated with a layer of gold. Imaging was performed using a Nova NanoSEM 200 scanning electron microscope using an intra-lens TLD detector with a beam voltage of 15 kV. The obtained images were analyzed using ImageJ software, segmenting the image into objects (corresponding to the outlines of grains) and analyzing the surface of the objects and their shape. The data on the grain surface size were then processed using the GrainSizeTools script (Python Programming Language) [42].

### 3.4. Determination of the Specific Surface and Characteristics of Porosity

Nitrogen adsorption isotherms were measured at −196 °C on the ASAP 2020 volumetric analyzer manufactured by Micromeritics Instrument Corp. (Norcross, GA, USA). All samples were outgassed at 200 °C for 2 h before adsorption measurements. The specific surface area (S_BET_) was estimated by means of the Brunauer–Emmett–Teller method [43]. By approximating the experimental data using the BET equation, the monolayer capacity was determined in the range of 0.05–0.25 relative pressures. The total pore volume was calculated using the volume of nitrogen adsorbed at p·p_0_^−1^ ≈ 0.99 [44]. The pore size distribution (PSD) functions were determined from nitrogen adsorption isotherms by using the non-local density functional theory method (2D-NLDFT) for carbon slit-shaped pores under the assumption of energetic heterogeneity and geometrical corrugation of the pore walls [45].

## 4. Results and Discussion

### 4.1. Crystal Structure

**Figure 2:**
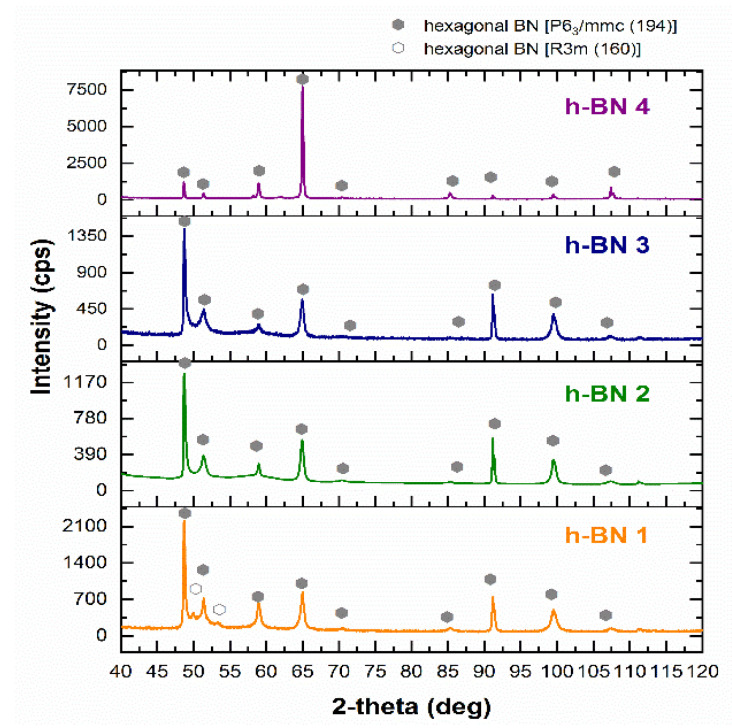
X-ray diffraction pattern of h-BN samples.

In all samples, by matching the appropriate reference XRD pattern, hexagonal boron nitride was identified (Figure 2). Detected h-BN types were described according to the international notation developed by Carl Hermann and Charles-Victor Mauguin. Sample h-BN 1 contained two types of hexagonal boron nitride with a different structure. Space group No. 194 represents a material with a typical hexagonal crystallographic structure, and space group No. 160 is a trigonal crystallographic system [46]. In this case, the trigonal system is considered a rhombohedral version of the hexagonal structure [47]. The radiation intensity recorded at particular values of the 2-theta angle was conditioned by the shape and particle size of h-BN. This can be seen especially when comparing the diffractogram obtained for sample h-BN 4 with the diffractograms of other samples. This fact is correlated with the differences in the shape and size of the particles of these samples examined by SEM. When comparing these diffractograms, it is also possible to notice the widening of the diffraction peaks for samples h-BN 1, h-BN 2, and h-BN 3 relative to sample h-BN 4. This results from the size of the crystallites. The similar course of diffraction patterns for the h-BN 2 and h-BN 3 samples in terms of the broadening of diffraction peaks and the intensity of X-ray radiation at certain 2-theta angle values indicates that there was a great similarity in the particle size of these h-BN types.

### 4.2. Quantitative and Qualitative Assessment of the Surface Composition

**Figure 3:**
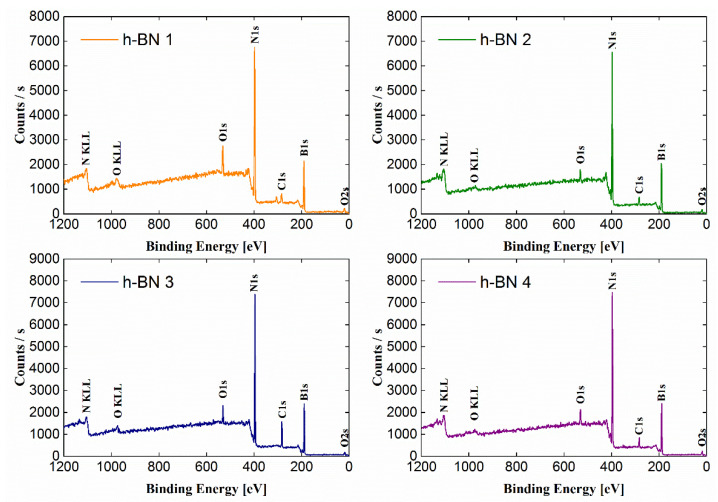
Wide-range spectra obtained for h-BN samples.

**Figure 4:**
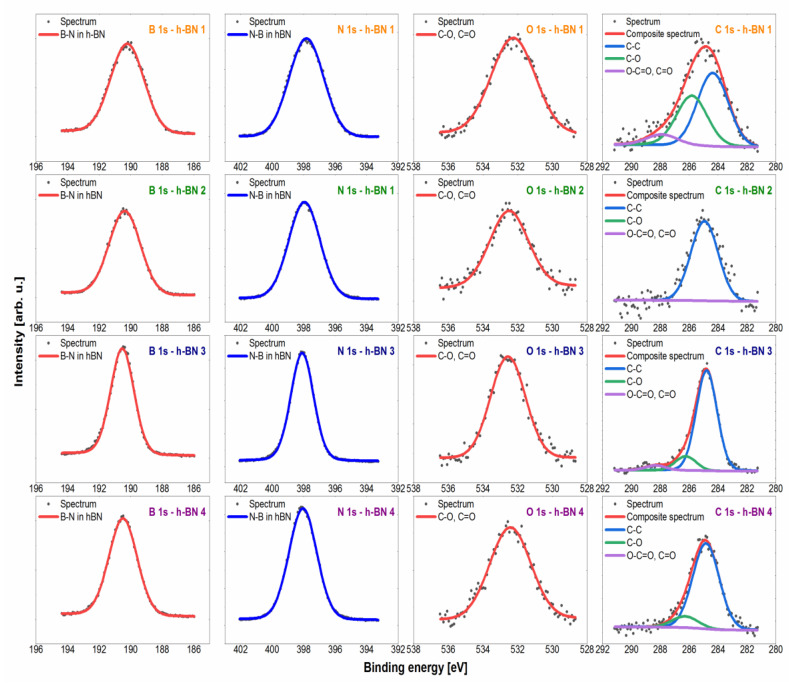
High-resolution spectra with fitted spectral lines for h-BN samples.

The wide-range low-resolution spectra for each tested sample obtained by the XPS method are presented in Figure 3. The most intense lines were identified and marked on the spectra. The atomic concentrations of the elements and the chemical bonds formed by them, determined based on the fit of the lines on the high-resolution spectra (Figure 4), are presented in Table 1. The atomic concentration of boron and nitrogen for all samples was similar and amounts to a total of 83.9–94.6 (% at.).

The spectra in the B 1s region for all samples tested were fitted with a single symmetric line with the maximum at the binding energy value of 190.3 eV, which confirms the presence of boron in hexagonal boron nitride [48]. In terms of the sensitivity of the XPS method (~0.5 at.%), there was no presence of both oxide species of type BNxOy, whose shifts were expected for the energy value of ~192 eV, and irregular forms of BNdis, which would be shown by a line for the energy value of ~189 eV [49]. The spectra collected in the N 1s region were fitted with one symmetrical line lying on the binding energy value of 397.9 eV, which corresponds to nitrogen in hexagonal boron nitride [48,49]. As in the case of the boron line, no other chemical states were observed that could be derived from oxidized or irregular forms. The spectrum in the C 1s region was fitted with 3 lines, of which line 1 with an energy value of 284.8 eV shows the C–C bond (sp^3^) [50,51], line 2 with an energy value of 286.3 eV corresponds to the presence of C–O–C and/or C–OH bonds [50,51], while line 3 lies at an energy value of 288.3 eV, which corresponds to O–C=O bonds [50]. The obtained carbon lines are characteristic of the XPS method. The carbon contamination formed in this method may be the result of deposition on the surface of a sample bombarded with an electron beam of the decomposition products of hydrocarbons and other organic compounds from the vacuum chamber or adsorbed on the tested surface [52]. The spectrum in the O 1s (oxygen) region was fitted with one line lying on the binding energy value of 532.5 eV, which corresponds to O=C and/or O–C bonds [50,51,53]. This line was associated with the presence of the aforementioned carbon contamination. Additionally, slight chemical shifts in the O 1s region for individual oxygen-containing compounds (both organic and inorganic) cause the overlapping of lines and the inability to unambiguously interpret the spectrum in this area.

### 4.3. Morphology and Particle Size Distribution

**Figure 5:**
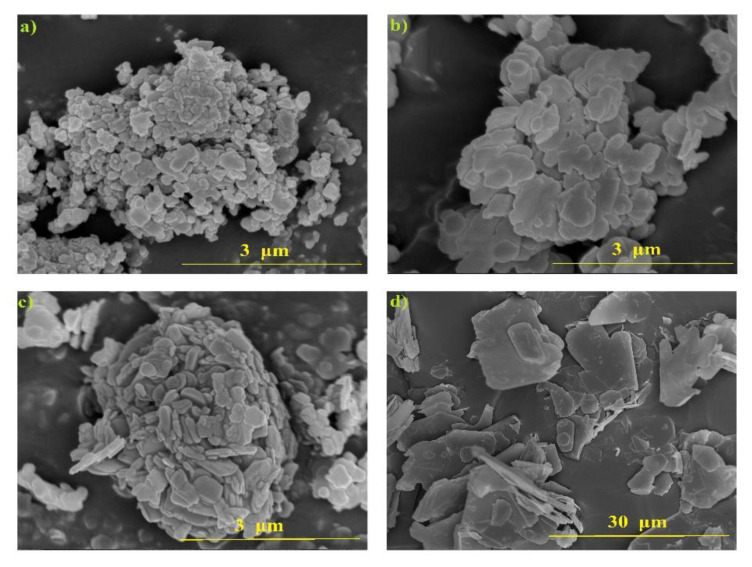
SEM picture of h-BN samples: (**a**) h-BN 1, (**b**) h-BN 2, (**c**) h-BN 3, and (**d**) h-BN 4.

The images of hexagonal boron nitride particles obtained with a scanning electron microscope are presented in Figure 5. The h-BN 2, h-BN 3 and h-BN 4 samples contained lamellar-shaped particles. The grain shape was non-isomeric, which should be considered when analyzing the results of the particle size composition research, based on the transformation of the particle surface into a circle and describing it by its diameter.

**Figure 6:**
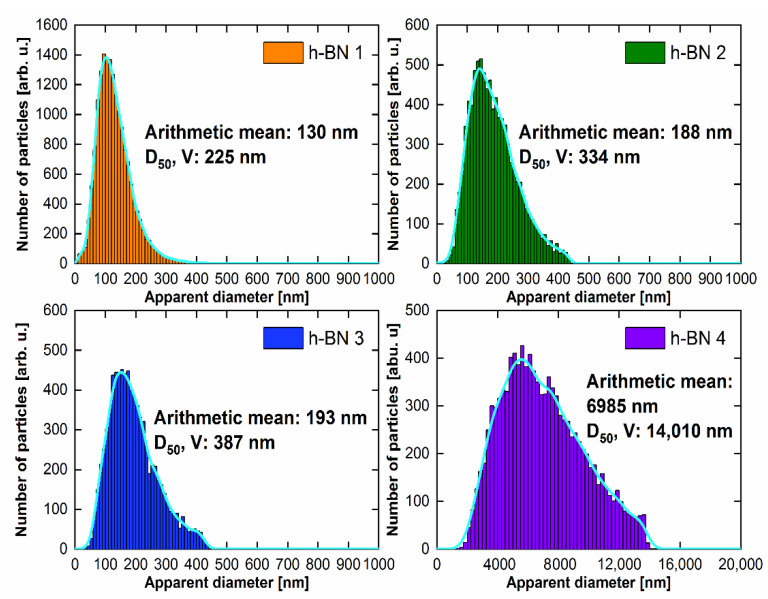
Particle size distribution for h-BN samples.

The particle size distribution histograms for the samples are shown in Figure 6. The surface area of each of the grains visible in the SEM photos was determined and then converted to the same area of a circle with an apparent diameter. The arithmetic mean of the set of such apparent diameters is given in the histograms. On the basis of microscopic observations, considering that the apparent diameter of the circle is the diameter of the sphere, the volume of each sphere was calculated and divided into size classes. After summing up the volumes of individual classes, the diameter D_50_ was calculated, for which smaller and larger grains account for half of the total volume. For each of the histograms, the data were smoothed using the kernel smoothing method (Silverman’s rule of thumb) [42].

The largest particle size was contained in the h-BN 4 sample. The statistical values that describe the particle diameter for the h-BN 2 and h-BN 3 samples are comparable. Sample h-BN 1 contained particles with the smallest diameters, of which a significant part can be classified as nanoparticles on this basis.

### 4.4. Specific Surface Area and Characteristics of Porosity

**Figure 7:**
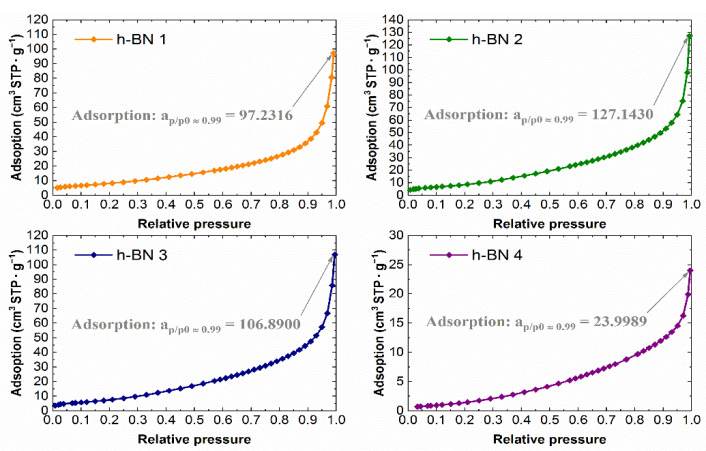
Nitrogen adsorption isotherms for h-BN samples.

**Figure 8:**
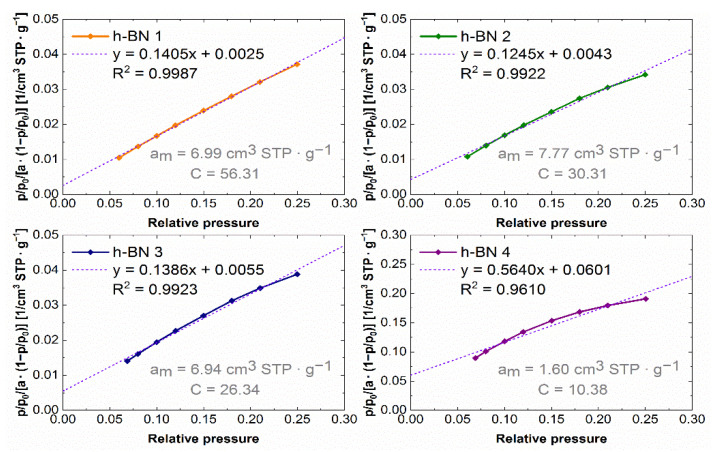
Nitrogen adsorption isotherms in BET equation coordinates for h-BN samples.

The experimental nitrogen adsorption isotherms obtained for individual samples are shown in Figure 7. They list the adsorption values, read for a relative pressure of ~0.99, from which the total pore volume values were calculated. The adsorption isotherms in the coordinates of the BET equation in the range of 0.05–0.25 relative pressure are shown in Figure 8. The values of the monolayer capacity a_m_ and the constant C, corresponding to the adsorption energy in the monolayer, are given. Both parameters were determined based on the coefficients of the linear equation, which are also given in Figure 8.

The values of the specific surface area and total pore volume for the h-BN samples are presented in Table 2.

**Figure 9:**
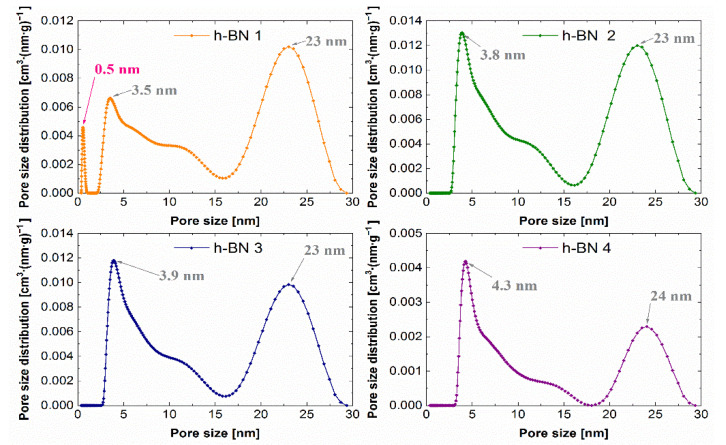
Pore size distribution functions for h-BN samples.

The lowest specific surface area, whose values together with the total pore volume are summarized in Table 2, which amounts to 7 m^2^·g^−1^, was observed for the h-BN 4. The total pore volume for this sample was also the lowest and was equal to 0.04 cm^3^·g^−1^. For samples of hexagonal boron nitride with a smaller particle size distribution (h-BN 1, h-BN 2, h-BN 3), similar values of the specific surface area and total pore volume were recorded. All the tested samples show heterogeneous pores, mainly in the area of mesopores. The pore volume distribution functions obtained using the above-mentioned methodology are presented in Figure 9.

The predominant pore sizes for individual samples were marked. The PSD functions determined for these samples allow the identification of two distinct peaks, indicating the predominant sizes of the mesopores of approximately 4 nm and 23 nm. Hexagonal boron nitride h-BN 1 has the smallest pores among the tested samples. Additionally, a low, sharp peak in the micropore region was observed for this sample.

### 4.5. The Analysis of the Properties in Terms of Lubrication Applications

The samples analyzed in the article contained hexagonal boron nitride, which was confirmed using X-ray diffraction and X-ray photoelectron spectrometry. Referring to the previously determined factors influencing the efficiency of lubrication, it should be assumed that the differences in properties between the various types of hexagonal boron nitride will result in their different effects in specific tribological systems. Sample h-BN 1 contained the smallest particles with an equivalent diameter D_50_ of 225 nm. It means that the contribution of particles included in the nano category distinguishes this sample from others. Therefore, it should be expected that the tribological interaction mechanisms characteristic for nanoparticles can be identified in the case of lubricants with the addition of h-BN 1. First, these particles can penetrate the target friction areas more efficiently and activate the mechanisms of surface protection mentioned above [7,18,35]. The particle size distribution determined for samples h-BN 2 and h-BN 3 was similar, while in the case of sample h-BN 4 the largest particles were identified. Therefore, it can be assumed, based on the experience of various authors [7,30], that such large particles may imply the worst tribological results. The specific surface area of the sample with the greatest granulation (h-BN 4) is the smallest, as is the total pore volume. The remaining samples are characterized by higher values of these parameters. The highest values were obtained for the h-BN 2 sample, and the h-BN 1 and h-BN 3 samples were characterized by similar values of the specific surface area and total pore volume. In publication [19], it was found that the developed pore structure may facilitate the binding of h-BN particles and a lubricant. In the case of our research, the h-BN 1, h-BN 2 and h-BN 3 samples are characterized by particles with a more developed porous structure (the h-BN 4 sample has a much smaller specific surface area and total pore volume). The combination of a more developed porous structure and a smaller size suggests that it may also be associated with an easier inflow of such particles into the friction zone. According to reports from the literature [4,9,10,11,12,13,14,16,17,18,19,20,22,23,24,27,29], the decisive stage should be expected to be the selection of the concentration of hexagonal boron nitride for a specific lubricant, ensuring the highest lubrication efficiency in the selected tribological system. The process of preparing lubricating oils with the addition of h-BN is a separate issue. The results of the tests carried out indicate the need to use chemical compounds and treatments to increase the stability of the suspension.

## 5. Conclusions

The analysis of the properties of hexagonal boron nitride enables the identification of the key factors that determine its use as an additive to lubricants. These include particle size distribution, their morphology, specific surface area and porosity. An important step is also selecting the appropriate concentration of h-BN particles in the lubricant. Understanding the properties of individual types of h-BN enables the identification of the mechanisms of their influence in tribological systems. The use of hexagonal nano boron nitride as an additive to lubricants seems to be promising due to its properties that allow for better cooperation of this type of particles in tribological systems compared to those of larger-sized particles.

## Figures and Tables

**Table 1:** Atomic concentrations (% at.) of elements with the division into the appropriate chemical environments.

Chemical Element	B	C			N	O
Binding energy (eV)	190.3	284.8	286.3	288.3	379.9	532.5
Chemical environment	h-BN	C–C	C–O	O–C=O	N–B	O–C O=C
h-BN 1	43.9	3.0	2.1	0.5	44.8	5.7
h-BN 2	45.7	2.9	0.0	0.0	48.9	2.5
h-BN 3	41.8	10.5	1.4	0.5	42.1	3.7
h-BN 4	45.3	4.8	0.6	0.0	46.2	3.1

**Table 2:** Specific surface area and total pore volume calculated for h-BN samples.

Sample	Specific Surface Area S_BET_ (m^2^·g^−1^)	Total Pore Volume V_t_ (cm^3^·g^−1^)
h-BN 1	30	0.15
h-BN 2	33	0.20
h-BN 3	30	0.16
h-BN 4	7	0.04

## Data Availability

The data presented in this study are available on request from the corresponding authors.
